# Supplementary material for: Cerebellar patients have intact feedback control that can be leveraged to improve reaching
Source: eLife. 2020 Oct 7;9:e53246. doi: 10.7554/eLife.53246 (PMC7577735; doi:10.7554/eLife.53246)
Supplement: Transparent reporting form [file elife-53246-transrepform.docx]

***eLife’s* transparent reporting form**

We encourage authors to provide detailed information *within their submission* to facilitate the interpretation and replication of experiments. Authors can upload supporting documentation to indicate the use of appropriate reporting guidelines for health-related research (see [EQUATOR Network](http://www.equator-network.org/%20)), life science research (see the [BioSharing Information Resource](https://biosharing.org/)), or the [ARRIVE guidelines](http://www.plosbiology.org/article/info:doi/10.1371/journal.pbio.1000412) for reporting work involving animal research. Where applicable, authors should refer to any relevant reporting standards documents in this form.

If you have any questions, please consult our Journal Policies and/or contact us: [editorial@elifesciences.org](mailto:editorial@elifesciences.org).

**Sample-size estimation**

- You should state whether an appropriate sample size was computed when the study was being designed
- You should state the statistical method of sample size computation and any required assumptions
- If no explicit power analysis was used, you should describe how you decided what sample (replicate) size (number) to use

Please outline where this information can be found within the submission (e.g., sections or figure legends), or explain why this information doesn’t apply to your submission:

When designing our study, we selected a large sample size: 31 subjects including 17 patients and 14 age-matched controls. This sample size is commensurate with similar studies in the human motor control literature and we had every expectation from previous studies that this sample size would produce statistically significant results if there were a biologically relevant effect.

**Replicates**

- You should report how often each experiment was performed
- You should include a definition of biological versus technical replication
- The data obtained should be provided and sufficient information should be provided to indicate the number of independent biological and/or technical replicates
- If you encountered any outliers, you should describe how these were handled
- Criteria for exclusion/inclusion of data should be clearly stated
- High-throughput sequence data should be uploaded before submission, with a private link for reviewers provided (these are available from both GEO and ArrayExpress)

Please outline where this information can be found within the submission (e.g., sections or figure legends), or explain why this information doesn’t apply to your submission:

-Table 1 shows how often each experiment was performed and the number of biological replicates.

-We did not include a definition for biological vs technical replication because we provided a clear explanation how many trials each subject undertook in Methods under Experimental Apparatus and Task Instructions. It is also clear to anyone in the field that biological replicates are individual participants while technical replicates are repeated trials, all of which are well described in the paper.

-The information in Table 1 combined with the aforementioned details in the -Methods section allow for clear understanding of the number of biological and technical replicates.

-One outlier was seen in Figure 3 and discussed in the Figure 3 caption. The standard criteria for outlier definition in a Matlab box and whisker plot was used to identify this outlier. The outlier is displayed and marked with an x.

-Criteria for exclusion/inclusion criteria are stated under Methods under Experimental Model and Subject Details

- We use no sequence data of this type in our submission

**Statistical reporting**

- Statistical analysis methods should be described and justified
- Raw data should be presented in figures whenever informative to do so (typically when N per group is less than 10)
- For each experiment, you should identify the statistical tests used, exact values of N, definitions of center, methods of multiple test correction, and dispersion and precision measures (e.g., mean, median, SD, SEM, confidence intervals; and, for the major substantive results, a measure of effect size (e.g., Pearson's r, Cohen's d)
- Report exact p-values wherever possible alongside the summary statistics and 95% confidence intervals. These should be reported for all key questions and not only when the p-value is less than 0.05.

Please outline where this information can be found within the submission (e.g., sections or figure legends), or explain why this information doesn’t apply to your submission:

- Statistical analysis methods are detailed under Methods under Experimental Design and Statistical Analysis

- The p-values are provided in the Results section for each experiment under the header for the relevant experiment.

(For large datasets, or papers with a very large number of statistical tests, you may upload a single table file with tests, Ns, etc., with reference to sections in the manuscript.)

**Group allocation**

- Indicate how samples were allocated into experimental groups (in the case of clinical studies, please specify allocation to treatment method); if randomization was used, please also state if restricted randomization was applied
- Indicate if masking was used during group allocation, data collection and/or data analysis

Please outline where this information can be found within the submission (e.g., sections or figure legends), or explain why this information doesn’t apply to your submission:

Our groups consisted simply of a patient group and a control group which were paired by age. The details of the groups are available under Methods under Experimental Model and Subject Details.

**Additional data files (“source data”)**

- We encourage you to upload relevant additional data files, such as numerical data that are represented as a graph in a figure, or as a summary table
- Where provided, these should be in the most useful format, and they can be uploaded as “Source data” files linked to a main figure or table
- Include model definition files including the full list of parameters used
- Include code used for data analysis (e.g., R, MatLab)
- Avoid stating that data files are “available upon request”

Please indicate the figures or tables for which source data files have been provided:

Data and code will be archived on the Johns Hopkins University Data Archive and will be assigned a unique DOI. The data will be available in the LIMBS Laboratory Dataverse: <https://archive.data.jhu.edu/dataverse/LIMBS>
